# Supplementary material for: A unique chromatin profile defines adaptive genomic regions in a fungal plant pathogen
Source: eLife. 2020 Dec 18;9:e62208. doi: 10.7554/eLife.62208 (PMC7781603; doi:10.7554/eLife.62208)
Supplement: Transparent reporting form [file elife-62208-transrepform.docx]

***eLife’s* transparent reporting form**

We encourage authors to provide detailed information *within their submission* to facilitate the interpretation and replication of experiments. Authors can upload supporting documentation to indicate the use of appropriate reporting guidelines for health-related research (see [EQUATOR Network](http://www.equator-network.org/%20)), life science research (see the [BioSharing Information Resource](https://biosharing.org/)), or the [ARRIVE guidelines](http://www.plosbiology.org/article/info:doi/10.1371/journal.pbio.1000412) for reporting work involving animal research. Where applicable, authors should refer to any relevant reporting standards documents in this form.

If you have any questions, please consult our Journal Policies and/or contact us: [editorial@elifesciences.org](mailto:editorial@elifesciences.org).

**Sample-size estimation**

- You should state whether an appropriate sample size was computed when the study was being designed
- You should state the statistical method of sample size computation and any required assumptions
- If no explicit power analysis was used, you should describe how you decided what sample (replicate) size (number) to use

Please outline where this information can be found within the submission (e.g., sections or figure legends), or explain why this information doesn’t apply to your submission:

We did not perform power analysis, sample size was determined based on sequencing feasibility. We utilized cross-sample analysis and multiple independent analysis methods to ensure our results are reliable.

**Replicates**

- You should report how often each experiment was performed
- You should include a definition of biological versus technical replication
- The data obtained should be provided and sufficient information should be provided to indicate the number of independent biological and/or technical replicates
- If you encountered any outliers, you should describe how these were handled
- Criteria for exclusion/inclusion of data should be clearly stated
- High-throughput sequence data should be uploaded before submission, with a private link for reviewers provided (these are available from both GEO and ArrayExpress)

Please outline where this information can be found within the submission (e.g., sections or figure legends), or explain why this information doesn’t apply to your submission:

The required information on replicates can be found in the materials and methods section under ‘Bisulfite sequencing and analysis’, ‘RNA-sequencing and analysis’, ‘Chromatin immunoprecipitation and sequencing and analysis’ and ‘ATAC-seq and analysis’.

Specifically, we used a single biological sample for the Bisulfite sequencing of individual genotypes and for ATAC-seq. We used three biological replicates for each of three growth conditions for RNA-seq (i.e. independent colonies grown in separate flasks of media, 3 reps * 3 media =9 samples). We used two biological replicates each for two ChIP-pull downs and sequencing. That is, two independent colonies were grown and used for independent pull downs using both H3K9me3 and H3K27me3 antibodies as detailed in materials and methods.

All sequencing information is deposited at NCBI SRA under the bioproject [PRJNA592220](https://www.ncbi.nlm.nih.gov/bioproject/PRJNA592220) as stated in the ‘Data availability’ section.

Information regarding analysis is provided in the indicated materials and methods sections.

**Statistical reporting**

- Statistical analysis methods should be described and justified
- Raw data should be presented in figures whenever informative to do so (typically when N per group is less than 10)
- For each experiment, you should identify the statistical tests used, exact values of N, definitions of center, methods of multiple test correction, and dispersion and precision measures (e.g., mean, median, SD, SEM, confidence intervals; and, for the major substantive results, a measure of effect size (e.g., Pearson's r, Cohen's d)
- Report exact p-values wherever possible alongside the summary statistics and 95% confidence intervals. These should be reported for all key questions and not only when the p-value is less than 0.05.

Please outline where this information can be found within the submission (e.g., sections or figure legends), or explain why this information doesn’t apply to your submission:

The statistical tests used for analysis are presented in each figure legend and in the corresponding materials and methods section on analysis. Non-parametric analysis were used throughout including Mann-Whitney U-test for pairwise comparisons, or Conover’s test for multiple sample comparisons. Any p-values shown in figures are exact p-values, and where symbols are used to signify statistical difference, the level is reported in the figure legend along with the exact p-value.

(For large datasets, or papers with a very large number of statistical tests, you may upload a single table file with tests, Ns, etc., with reference to sections in the manuscript.)

**Group allocation**

- Indicate how samples were allocated into experimental groups (in the case of clinical studies, please specify allocation to treatment method); if randomization was used, please also state if restricted randomization was applied
- Indicate if masking was used during group allocation, data collection and/or data analysis

Please outline where this information can be found within the submission (e.g., sections or figure legends), or explain why this information doesn’t apply to your submission:

Samples were not analyzed or allocated into groups.

**Additional data files (“source data”)**

- We encourage you to upload relevant additional data files, such as numerical data that are represented as a graph in a figure, or as a summary table
- Where provided, these should be in the most useful format, and they can be uploaded as “Source data” files linked to a main figure or table
- Include model definition files including the full list of parameters used
- Include code used for data analysis (e.g., R, MatLab)
- Avoid stating that data files are “available upon request”

Please indicate the figures or tables for which source data files have been provided:

The DNA and RNA sequencing data used throughout the analysis is publicly available through the NCBI SRA bioproject [PRJNA592220](https://www.ncbi.nlm.nih.gov/bioproject/PRJNA592220). Additional data files are provided as supplementary information. Parameters used for individual programs are analysis details are provided in the analysis sections of the materials and methods.
